# The artificial meal SkitoSnack does not support reproduction in *Culex pipiens* (Diptera: *Culicidae*) mosquitoes

**DOI:** 10.1093/jisesa/ieaf022

**Published:** 2025-04-25

**Authors:** Alina Soto, Ann-Sophie Devlies, Lotte Wauters, Ana Paula Ferreira Pinto, Leen Delang

**Affiliations:** Virus-Host Interactions & Therapeutic Approaches (VITA) Research Group, Department of Microbiology, Immunology and Transplantation, Rega Institute for Medical Research, KU Leuven, Leuven, Belgium; Virus-Host Interactions & Therapeutic Approaches (VITA) Research Group, Department of Microbiology, Immunology and Transplantation, Rega Institute for Medical Research, KU Leuven, Leuven, Belgium; Virus-Host Interactions & Therapeutic Approaches (VITA) Research Group, Department of Microbiology, Immunology and Transplantation, Rega Institute for Medical Research, KU Leuven, Leuven, Belgium; Virus-Host Interactions & Therapeutic Approaches (VITA) Research Group, Department of Microbiology, Immunology and Transplantation, Rega Institute for Medical Research, KU Leuven, Leuven, Belgium; Virus-Host Interactions & Therapeutic Approaches (VITA) Research Group, Department of Microbiology, Immunology and Transplantation, Rega Institute for Medical Research, KU Leuven, Leuven, Belgium

**Keywords:** artificial meal, life history, mosquito colony, rearing

## Abstract

Mosquitoes are hematophagous insects. Obtaining fresh animal blood to maintain laboratory colonies, rear high numbers of mosquitoes, or blood-feed mosquitoes for experimental purposes, can be costly and imposes ethical concerns. Recently, the artificial meal SkitoSnack was developed to rear *Aedes aegypti* L. mosquitoes. This artificial diet is low-cost, can be easily prepared in the laboratory, and results in comparable life history traits to *Ae. aegypti* raised with animal blood. Here, we investigated if the SkitoSnack can be used to produce the next generation of *Culex pipiens* L. as a substitute for animal blood and assessed the effects on mosquito fitness. Female *Cx. pipiens* fed with SkitoSnack demonstrated high post-feeding mortality and lower fecundity, fertility, egg-laying rates, egg-hatching rates, and offspring emergence rates compared to those fed with vertebrate animal blood. In contrast, the longevity and body sizes of the offspring were not significantly different between the 2 feeding groups, suggesting that the first generation of SkitoSnack-reared mosquitoes had similar fitness to those raised from animal blood. Feeding a different generation of *Cx. pipiens* resulted in a similar loss of fitness in the SkitoSnack-fed females; however, these females were unable to produce viable offspring. In addition, we fed the SkitoSnack to *Ae. aegypti*, which also resulted in a significant reduction in fecundity and fertility. A significant loss of life and reproductive capacity was observed in SkitoSnack-fed *Cx. pipiens*, but more research is required to determine whether optimizing the current SkitoSnack formula can improve the fitness outcomes of fed females.

## Introduction

Mosquitoes are hematophagous insects that require a fresh blood meal to produce and lay eggs. After a singular mating event, female mosquitoes must locate and feed on a suitable vertebrate host, seek and rest in a sheltered location to complete follicular development, and locate a suitable aquatic habitat to lay a batch of eggs (Clements 1992). This gonotrophic cycle is repeated until the end of life. Some mosquito species can produce a first batch of eggs without a blood meal (autogeny), although these egg batches are usually smaller and a blood meal is required to produce subsequent egg batches (Awahmukalah and Brooks 1985). Mosquitoes display unique species- and sub-species-specific differences for the timing of host-seeking (diurnal vs. nocturnal), indoor or outdoor host-seeking behavior (endophagy vs. exophagy), and source (mammalian, avian, and reptilian) for blood meals. Vertebrate blood, which contains the necessary proteins and iron required for successful egg production, varies greatly depending on the source (Harrison et al. 2021). The quality, quantity, and source of blood meals can majorly alter the reproduction and fitness of mosquitoes (Richards et al. 2012), as well as their ability to transmit human and animal pathogens (Harrison et al. 2021, Johnson et al. 2023). For this reason, mosquitoes typically display a strong preference toward one or several specific host species. This is evident by the *Culex pipiens* L. (Diptera: Culicidae) biotypes, which differ significantly in their host-seeking behavior. *Cx. pipiens* (the Northern house mosquito) is divided into 2 biotypes: *Cx. pipiens pipiens* and *Cx. pipiens molestus*. The *pipiens* biotype is anautogenous and displays a preference for avian hosts, whereas the *molestus* biotype is autogenous and prefers to obtain blood meals from mammals.

Laboratory colonies of mosquitoes are needed in research and to produce large quantities of mosquitoes for control strategies. They are typically maintained by allowing mosquitoes to directly feed on live animals or human volunteers, or by delivering a pre-prepared blood meal from an animal source through an artificial feeding system. Artificial membrane feeders warm the blood and allow mosquitoes to feed through a membrane (Gonzales and Hansen 2016) or other means such as parafilm jackets (Finlayson et al. 2015, Sri-in et al. 2020) or sausage casings (Deng et al. 2012). The use of live animals such as mice, rabbits, and guinea pigs—that are often sedated prior to feeding—imposes ethical concerns (Ferdowsian and Beck 2011) as well as the need for specialized housing and care, trained personnel, and compliance with the policies of institutional and/or regional ethical committees. The same requirements can apply to obtaining fresh blood from animals, as well as the need to process the blood to remove anticoagulants (washing) (Adams et al. 2021) or add phagostimulants such as adenosine triphosphate (ATP) or sugars to enhance blood-feeding rates (Hosoi 1959, Galun 1967). These rearing strategies can be rather costly over time when regular blood-feeding is required to maintain colonies. Furthermore, animal blood has a short shelf life (up to 2 weeks), requires constant cooling, and blood meal composition may be inconsistent between batches due to different animal sources and varying use of anticoagulants (Richards et al. 2012, Gonzales and Hansen 2016).

A blood-free alternative that is standardized, simple to prepare, low-cost, and pathogen-free would eliminate the need for live animals and fresh animal blood. To replace vertebrate blood, females must readily ingest the artificial meal in sufficient amounts, the meal must support egg development and production of large egg batches, the competitiveness (fitness) of offspring should be comparable to those of blood-fed mosquitoes, and mosquito behavior and immunity should not be affected (Gonzales et al. 2015, Gonzales and Hansen 2016). Several studies have investigated the use of artificial diets or modified blood meals for the maintenance of mosquitoes (Dimond et al. 1955, Lea et al. 1955, Kogan 1990, Griffith and Turner 1996, Jason Pitts 2014, Gonzales et al. 2015, 2018, Talyuli et al. 2015, Baughman et al. 2017, Marques et al. 2020). The meals usually contain a mixture of proteins such as γ-globulins as the key nutrients for egg development, salts such as NaCl, NaHCO_3_, and KCl to act as pH buffers, and phagostimulants to stimulate feeding. Baughman et al. (2017) established a modified blood mixture consisting of human plasma and ATP as an effective alternative to blood, with a long storage time in cold conditions (Baughman et al. 2017). In addition, Talyuli et al. (2015) indicated the importance of dietary cholesterol, as mosquitoes do not produce this themselves (Talyuli et al. 2015). The cholesterol reserve affects the reproductive performance of female offspring, partially due to its role in the production of oogenesis-regulating hormones. Most of these studies were conducted using Aedine and Anopheline mosquitoes, as they are the main vectors for human pathogens including *Plasmodium* parasites and mosquito-borne viruses of public health concern. The only study that tested a blood meal substitute in *Culex* mosquitoes was by Griffith and Turner (1996). The artificial meal consisted of proteins including globulins and ovalbumin, soya infant formula, and ATP. The diet was able to successfully maintain a colony of *C. quinquefasciatus* for over 50 generations and resulted in similar life history traits to the control group fed on live guinea pigs. However, few fitness parameters were evaluated (fertility, mortality, reproductivity, and adult weight). It would be interesting to evaluate the impact of artificial meals on rearing *Cx. pipiens*, as they are considered the primary vector for mosquito-borne viruses in the northern hemisphere, including West Nile virus, Usutu virus, St. Louis Encephalitis virus, avian malaria, and canine worms (Schulz and Becker 2018).

An artificial meal called the SkitoSnack was developed by Gonzales et al. (2018). This meal is a mixture of proteins, phagostimulants, and salts dissolved in water and warmed to 37 °C (Gonzales et al. 2018). *Aedes aegypti* L. (Diptera: Culicidae) and *Aedes albopictus* fed with SkitoSnack for 10 or more generations had similar life history traits (fecundity, egg-laying, hatch rates, body weight, and wing length) as blood-fed mosquitoes. Offspring from SkitoSnack-fed females showed analogous infection and dissemination rates of dengue virus type 2 (DENV-2), but a significant reduction of infection with DENV-4, when compared to blood-fed mosquitoes. The SkitoSnack is estimated to cost $0.22 per milliliter and can be stored for at least 3 months in its solid form at −20 °C. Due to species-specific differences in blood-feeding, our aim was to evaluate if the SkitoSnack could successfully produce a second generation of a laboratory colony of *Cx. pipiens pipiens* with comparable outcomes to blood-fed mosquitoes. Compared to a control group fed with vertebrate animal blood, we evaluated SkitoSnack feeding rates, oviposition (egg-laying) rates, fecundity (number of eggs per female), fertility (number of larvae per female), pupal development, and offspring longevity and body size. In addition, we included *Ae. aegypti* mosquitoes as a control group to evaluate any species-specific effects caused by the SkitoSnack.

## Methods

### Mosquitoes

*Cx. pipiens* biotype *pipiens* mosquitoes were provided by Prof. Sander Koenraadt (Wageningen University & Research, Wageningen, Netherlands). This colony originated from Brummen, The Netherlands (°05′23.2″N 6°09′20.1″E) and was established in 2010 (Fros et al. 2015). In our insectary facility, eggs were hatched in plastic HDPE trays (350 × 235 × 73 mm, Novolab) containing 2 L water. Larvae were fed daily on Superlevure yeast tablets (Gayelord Hauser, Saint-Genis-Laval, France) and TetraMin baby fish food (Tetra, Melle, Germany). Pupae were collected in water and placed in BugDorm-4S3030 Insect Rearing cages (Watkins & Doncaster, Leominster, United Kingdom). Mosquito larval, pupal, and adult stages were maintained at 25 °C with 70% relative humidity (RH) on a 16:8 h light:dark cycle. Adults were provided 10% sugar dissolved in Milli-Q water (Merck-Millipore, Burlington, USA) ad libitum. To maintain the mosquito colony in the laboratory, female mosquitoes were fed a mixture of chicken blood and fetal bovine serum (FBS), after which they were allowed to lay eggs. The first generation of this colony reared in our insectary facility was designated as F_0_.

*Ae. aegypti* belonging to the Paea strain (Papeete, Tahiti, 1994) were obtained from Prof. A. Failloux via the Infravec2 consortium. Eggs were hatched in dechlorinated tap water and larvae were transferred to plastic trays containing 3 L of water and fed daily with a yeast tablet (Gayelord Hauser). Pupae were placed in small bowls and allowed to emerge inside BugDorm cages. Adult mosquitoes were provided with 10% sugar-soaked cotton pledgets ad libitum and females were blood-fed with rabbit blood to support egg production. The trays and cages were incubated at 28 °C and 80% RH with a 16:8 h light:dark cycle.

### SkitoSnack Preparation

The SkitoSnack was prepared following the methods described by Gonzales et al. (2018). In brief, the SkitoSnack components (Table 1) were weighed individually and aliquots of dry compounds for 15 ml of SkitoSnack were prepared and stored at 4 °C until the day of feeding. All compounds were obtained from Sigma-Aldrich (Saint Louis, USA) with the exception of ATP, NaHCO_3_, and KCl, which were obtained from Cayman Chemical (Ann Arbor, USA), Honeywell Fluka Chemicals (Morris Plains, USA), and Acros Organics (Geel, Belgium), respectively. Prior to feeding, the compound mixture was dissolved in 15 ml Milli-Q water, vortexed thoroughly, and placed in a 37 °C water bath until a homogenous brown-colored solution was obtained.

**Table 1.** Reagents and their final concentrations in the SkitoSnack formula (Gonzales et al. 2018)

Component	Concn	Role
Bovine serum albumin (BSA)	200 mg/ml	Macronutrient (protein)
Bovine hemoglobin	5 mg/ml	Iron
Chicken yolk	5 mg/ml	Macronutrient (lipid, protein)
Glucose	50 mM	Phagostimulant
Adenosine triphosphate (ATP)	3 mM	Phagostimulant
Sodium chloride (NaCl)	150 mM	Buffer
Sodium bicarbonate (NaHCO_3_)	23 mM	Buffer
Potassium chloride (KCl)	4 mM	Buffer
Calcium chloride (CaCl_2_)	2.5 mM	Buffer
Magnesium chloride (MgCl_2_)	0.8 mM	Buffer

### Blood Meal Preparation

*Cx. pipiens pipiens* were fed with a mixture of chicken blood and FBS while *Ae. aegypti* were fed with rabbit blood. On the day of feeding, 10 ml of chicken blood was collected in 50 ml tubes with 50,000 UI/ml of heparin dissolved in 10 ml of phosphate-buffered saline (PBS). The heparin was removed by centrifugation (4,000 rpm for 3 min), followed by 3 washing and centrifugation steps with PBS (3,000 rpm for 2 min each step). The pellet of blood was resuspended in FBS at a 2:1 ratio of blood to FBS. The blood and FBS mixture was kept at 4 °C until feeding. The rabbit blood was collected in 15-ml tubes containing 500 UI/ml of heparin dissolved in 100 µl of PBS and kept at 4 °C until feeding. Immediately prior to feeding, 5 mM of ATP was added to the chicken and rabbit blood meals.

### *Culex* Feeding

For the experiments, *Cx. pipiens pipiens* belonging to the F_4_ or the F_13_ generation were used. A total of 3 independent replicates were conducted using the F_4_ group. The experiments were repeated once with an older generation (F_13_) to examine if the results would be significantly different when using a different generation. For each replicate, fresh chicken blood was obtained and resuspended in FBS as described above and a new aliquot of SkitoSnack was used. Mosquitoes were collected in cardboard cups and sugar-starved for 24 h prior to feeding. The cups were kept in an incubator at 25 °C and 70% humidity. A Hemotek (Blackburn, UK) feeding system was set at 37 °C. In each feeder, the meals were added to a total volume of 3 ml per feeder using parafilm (Parafilm M laboratory wrapping film; Bemis, Neenah, WI, USA) as the feeding membrane. The mosquitoes were prepared for feeding by switching off the light approximately 30 min prior to feeding and were fed between 17:00 and 19:00 for 30 min. Fed females were sorted from unfed mosquitoes over dry ice immediately after feeding and were placed in separate cages.

### *Aedes* Feeding

*Ae. aegypti* (Paea strain; InfraVec consortium) were fed following the same feeding protocol as the *Culex* mosquitoes but with minor differences. Fresh rabbit blood was obtained and a new aliquot of SkitoSnack was used. Mosquitoes were sugar-starved for 24 h prior to feeding in cardboard cups and kept at 28 °C and 80% humidity. A Hemotek (Blackburn, UK) feeding system was set at 37 °C using a thin collagen membrane (Hemotek feeding membrane) as the feeding membrane. In each feeder, the meals were added to a total volume of 3 ml per feeder. The mosquitoes were fed in the morning at 11:00 for 30 min. Fed females were sorted from unfed mosquitoes over ice immediately after feeding and were placed in separate cages.

### Life History Trait Evaluation

Feeding rates were calculated based on the number of fed mosquitoes obtained during each feeding replicate (Table 2). Digestion was determined by visually comparing the abdomens of mosquitoes on the day of feeding under a stereomicroscope (VisiScope, VWR, Radnor, USA). Mosquito mortality after feeding was determined by daily monitoring of the number of dead mosquitoes. Fecundity was determined at 9 days (*Cx. pipiens pipiens*) or 10 days (*Ae. aegypti*) after feeding by dissecting the ovaries of cold-anesthetized mosquitoes in PBS and counting the number of eggs per female. To determine oviposition rates, eggs were collected for up to 7 days, starting 9–10 days after feeding (after females were sampled for ovary dissection). *Cx. pipiens pipiens* were given a small glass bowl filled with tap water for laying egg rafts, while *Ae. aegypti* were individually placed in oviposition chambers as described by Gonzales et al. (2018). One *Cx. pipiens pipiens* egg raft represented one female that laid a batch of eggs, but the eggs were not counted due to their clustering which made it difficult to count accurately. *Aedes* females were allowed to lay their eggs on damp parchment paper, after which the papers were dried for 3 days at 28 °C and counted under a stereomicroscope (VisiScope, VWR) prior to hatching. The number of mosquitoes that deposited eggs was then compared to the number of live mosquitoes on the first egg-collection day. For the calculation of fertility in *Cx. pipiens pipiens*, individual egg rafts were transferred to plastic boxes filled with tap water. For *Ae. aegypti*, papers with dried eggs were transferred to cardboard cups filled with tap water. The exact number of larvae per female was counted by transferring each larva to plastic HDPE trays filled with tap water (350 × 235 × 73 mm, Novolab). An estimated hatch rate was calculated for the *Culex* eggs based on the average fecundity and fertility values, whereas the exact hatch rate was determined for the *Aedes* group. To determine the larval development time, the emergence of new pupae wase counted daily since the date the eggs were laid (*Culex*) or submerged in water (*Aedes*). The pupation rate was determined by comparing the number of pupae to the number of larvae in each tray. Pupae were transferred to small water-containing bowls, and placed in cages to allow adult emergence. Longevity of the *Cx. pipiens pipiens* F_1_ offspring was measured by counting daily the number of dead mosquitoes in the cage. A 10% sugar solution was continuously provided to the mosquitoes in the longevity assay. To measure adult wing sizes, 2- to 5-day-old mosquitoes were cold-anesthetized and placed on a Petri dish. The left wing was carefully removed from the thorax using superfine stainless-steel forceps (Watkins & Doncaster) and placed on a stage micrometer (Carl Zeiss, Oberkochen, Germany) in silicone oil (Sigma-Aldrich). A stereomicroscope was used to visualize and measure the wing length from the apical notch to the axillary margin, excluding the wing fringe (Yeap et al. 2013).

**Table 2.** Outcomes evaluated per mosquito subject group

Subject	Outcome	Calculation
Parent (fed)	Feeding rate	*n* fed / *n* total × 100
	Post-feeding mortality	*n* days from feeding to death
	Fecundity	*n* eggs / fed female
	Oviposition (egg-laying) rate	*n* fed females that laid eggs / *n* fed females x 100
	Fertility (egg-hatching)	*n* larvae / fed female
	Estimated hatch rate (*Culex*)	(Mean larvae / fed female) / (mean eggs / fed female) × 100
	Hatch rate (*Aedes*)	*n* larvae / *n* eggs laid × 100
Offspring (unfed)	Larval development (*Culex*)	*n* days from egg-laying to pupae
	Larval development (*Aedes*)	*n* days from egg submergence to pupae
	Pupation rate	*n* pupae / *n* larvae × 100
	Adult emergence rate	*n* adults / *n* pupae × 100
	Adult longevity (*Culex*)	*n* days from adult emergence to death
	Adult size (wing length)	Mean wingspan (mm)

### Data Analysis and Presentation

All figures and statistical analyses were performed with GraphPad Prism v10.2.0 (GraphPad Software, San Diego, USA) (GraphPad Prism 10 User Guide). Normality was determined using the Shapiro–Wilk test. A *P*-value of < 0.05 was considered statistically significant.

### Ethics Statement

Animal blood was obtained under the approval of the Ethical Committee of KU Leuven (license P150/2018) following institutional guidelines approved by the Federation of European Laboratory Animal Science Associations (FELASA).

## Results

### The Effects of the SkitoSnack on *Culex pipiens pipiens*

**Fig. 1.**
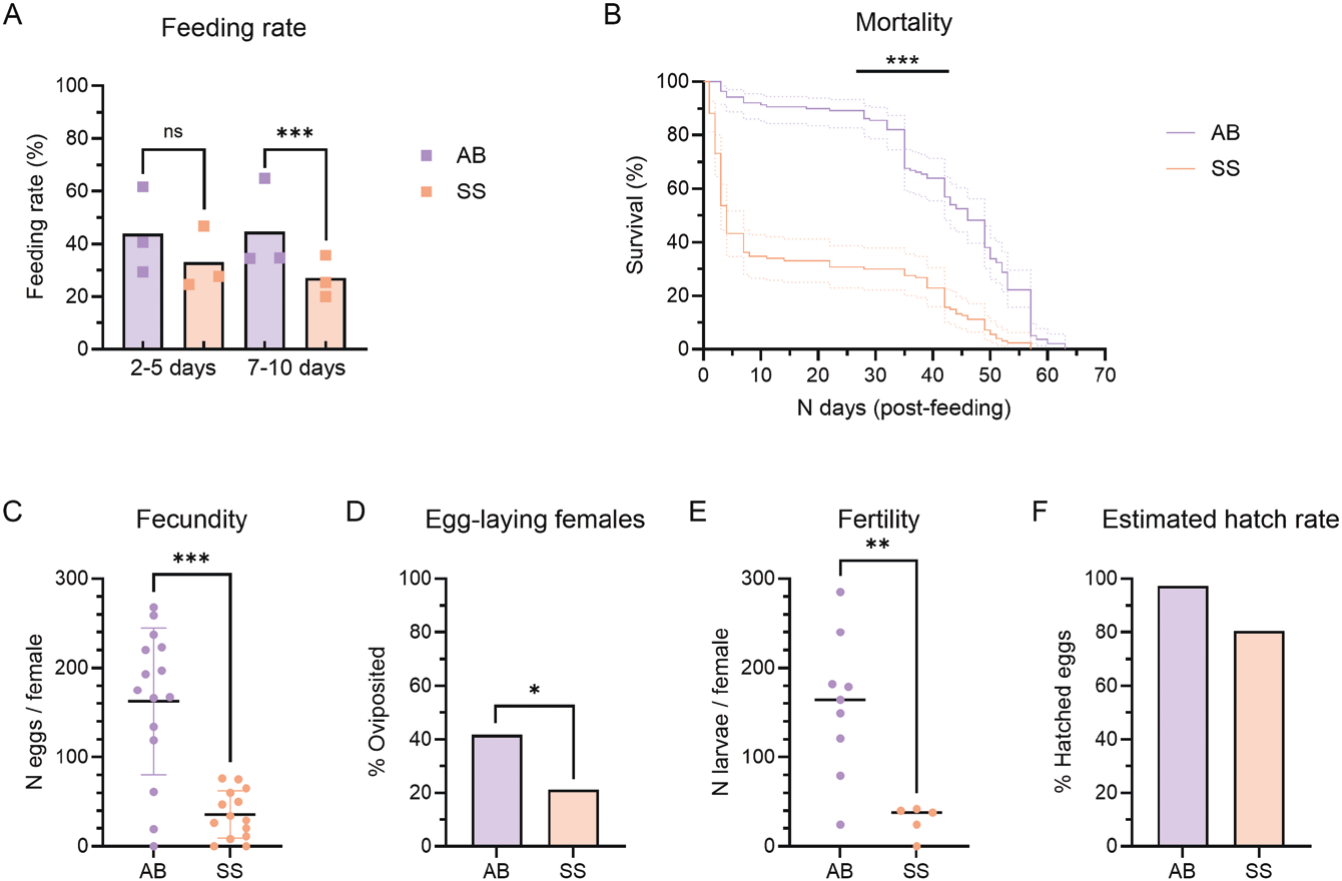
Feeding, survival, and reproduction of F_4_*Cx. pipiens pipiens* fed with SkitoSnack (SS) or animal blood (AB). A) Feeding rates of females from different age groups offered the SkitoSnack (2–5 days: *n* = 235; 7–10 days: *n* = 219) or animal blood (2–5 days: *n* = 228; 7–10 days: *n* = 231). The colored squares represent individual feeding experiments; the bars represent the mean feeding rate. Fisher’s exact test; ns: non-significant; ***: *P* ≤ 0.001. B) Mortality of fed females (SkitoSnack: *n* = 127; animal blood: *n* = 139). The dotted lines represent the 95% confidence intervals. Log-rank (Mantel–Cox) test; ***: *P* ≤ 0.001. C) Fecundity measured as the total number of eggs per female (SkitoSnack: *n* = 14; animal blood: *n* = 15). The dots represent individual females; the black line represents the mean; the colored lines represent the standard deviation. Unpaired *t* test; ***: *P* ≤ 0.001). D) Proportion of females that laid a batch of eggs (SkitoSnack: *n* = 33; animal blood: *n* = 127). Fisher’s exact test; *: *P* ≤ 0.05. E) Fertility measured as n larvae per egg raft / female (SkitoSnack: *n* = 5; animal blood: *n* = 9). The colored dots represent individual egg rafts; the black lines represent the median. Mann–Whitney test; **: *P* ≤ 0.01. F) Proportion (%) of hatched eggs estimated from fecundity and fertility data.

Sugar-starved females belonging to 2 age groups (2–5 or 2–10 days old) were offered either the SkitoSnack or animal blood for feeding (Fig. 1A). The feeding rate was not significantly different for 2- to 5-day-old females (SkitoSnack: 33.0%; animal blood: 43.9%; Fisher’s exact test, *P* > 0.05), but was significantly lower in the older age group fed with SkitoSnack compared to animal blood (SkitoSnack: 27.0%; animal blood: 44.7%; Fisher’s exact test, *P* ≤ 0.001). A total of 131 and 191 F_4_ females were fed in the SkitoSnack and animal blood control groups, respectively. Ingestion of the SkitoSnack was visually apparent based on the engorgement of the abdomen immediately after feeding and the gradual appearance of eggs over 48 hours (Supplementary Fig. S1). Following feeding, the fed females of both age ranges (2–5 and 7–10 days old) were pooled together to monitor mortality. Mortality was significantly higher in the SkitoSnack group (Fig. 1B), with a median survival rate of 4 days post-feeding, whereas those fed with animal blood had a median survival of 46 days [Log-rank (Mantel–Cox) test; *P* ≤ 0.001]. Fecundity was significantly lower in the SkitoSnack group [Fig. 1C; unpaired *t* test (*t* = 5.481; *df* = 27; *P* ≤ 0.001)]. The mean (± SD) eggs per female was 36 ± 27 in the SkitoSnack group and 163 ± 82 in the animal blood group. In addition, when the eggs were examined microscopically, several females in the SkitoSnack group had eggs that did not reach complete maturation, whereas underdeveloped eggs were not observed in the animal blood group (Supplementary Fig. S2). The proportion of females that laid eggs (Fig. 1D) was also significantly lower when females fed on the SkitoSnack compared to animal blood (SkitoSnack: 21%; animal blood: 42%; Fisher’s exact test; *P* ≤ 0.05). Of the females that laid a batch of eggs, the median number of larvae per female (fertility rate) was 164 and 38 in the animal blood and SkitoSnack groups, respectively (Fig. 1E). Fertility was thus significantly lower in the SkitoSnack group (Mann–Whitney test; *P* ≤ 0.01). As it is difficult to individually count the number of eggs clustered in each egg raft, the egg hatch rate was estimated based on the fecundity and fertility data (Fig. 1F). The estimated hatch rate was 97.3% in the animal blood group and 80.5% in the SkitoSnack group.

**Fig. 2.**
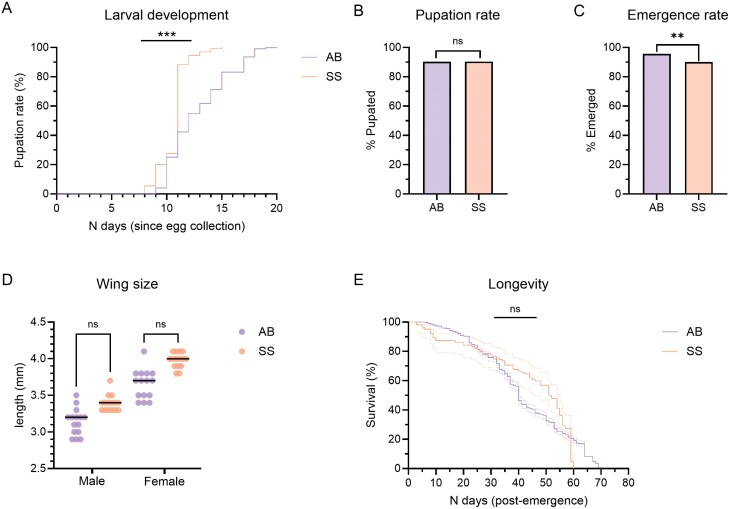
Fitness of *Cx. pipiens pipiens* offspring from F_4_ parents fed with SkitoSnack (SS) or animal blood (AB). A) Rate of pupation (%) over number of days since egg rafts were laid (SkitoSnack: *n* = 130; animal blood: *n* = 1,276). Log-rank (Mantel–Cox) test; ***: *P *≤ 0.001. B) Proportion (%) of pupae that molted from larvae (SkitoSnack: *n* = 144; animal blood: *n* = 1,415). Fisher’s exact test; ns: non-significant. C) Proportion (%) of adults that emerged from pupae (SkitoSnack: *n* = 130; animal blood: *n* = 1,266). Fisher’s exact test; **: *P* ≤ 0.01. D) Wingspan (mm) of males and females (*n* = 15 per group). The colored dots represent individual values; the black lines represent the median. Kruskal–Wallis test; ns: non-significant. E) Longevity (*n* days) from adult emergence to death (SkitoSnack: *n* = 102; animal blood: *n* = 1,242). The dotted lines represent the 95% confidence intervals. Log-rank (Mantel–Cox) test; ns: non-significant.

Larval development time from hatching to pupation (Fig. 2A) was shorter in the SkitoSnack group, ranging from 8 to 15 days (median: 11 days) compared to a range of 9–20 days (median: 12 days) in the animal blood group [Log-rank (Mantel–Cox) test; *P**** ***≤ 0.001]. The pupation rate (Fig. 2B) was not significantly different between the SkitoSnack (90.3%) and animal blood (90.2%) groups (Fisher’s exact test; *P* > 0.05). The rate of adult emergence (Fig. 2C) was significantly lower in the SkitoSnack group, although the difference was modest compared to the animal blood group (90.0% and 95.7%, respectively; Fisher’s exact test; *P* ≤ 0.01). The wing sizes of emerged offspring were not significantly different between the males or females of the 2 feeding groups [Fig. 2D; Kruskal–Wallis test, *P* > 0.05 (males), *P* > 0.05 (females)]. Males had a median wingspan of 3.2 and 3.4 mm and females had a median wingspan of 3.7 and 4.0 mm in the animal blood and SkitoSnack groups, respectively. The longevity of the SkitoSnack group was also not significantly different from the animal blood group [Fig. 2E; Log-rank (Mantel–Cox) test, *P* > 0.05]. The median lifespan of the offspring was 52 and 40 days in the SkitoSnack and animal blood groups, respectively.

### The Effects of the SkitoSnack on an Older Generation (F_13_) of *Culex pipiens pipiens*

**Fig. 3.**
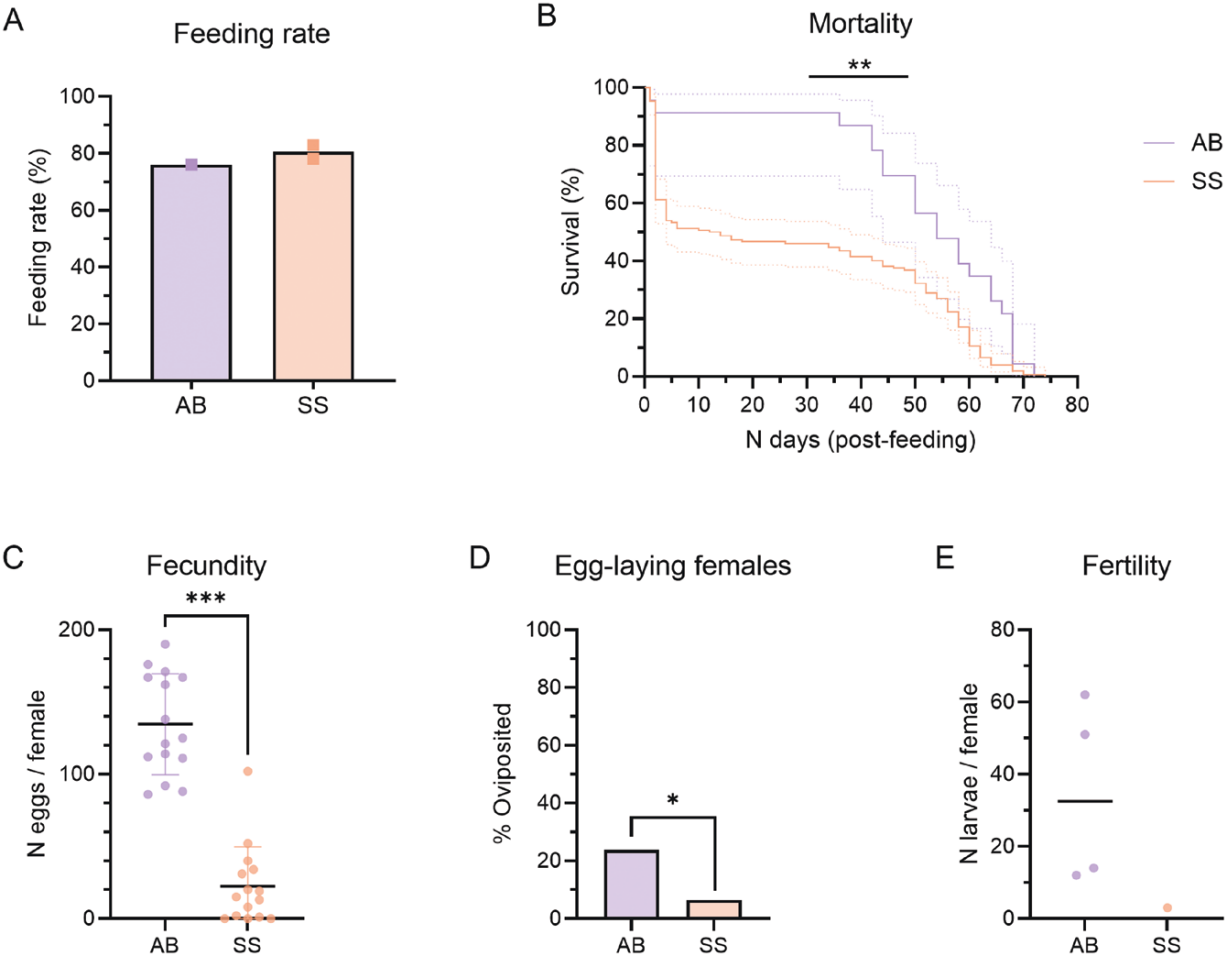
Feeding, survival, and reproduction of F_13_*Cx. pipiens pipiens* fed with SkitoSnack (SS) or animal blood (AB). A) Feeding rates of females offered the SkitoSnack (*n* = 208) or animal blood (*n* = 50). The bars represent the mean feeding rate of 1 (animal blood) or 2 (SkitoSnack) feedings. B) Mortality of fed females (*n* = 152; animal blood: *n* = 23). The dotted lines represent the 95% confidence intervals. Log-rank (Mantel–Cox) test; **: *P* ≤ 0.01. C) Fecundity measured by the total number of eggs per female (SkitoSnack: *n* = 15; animal blood: *n* = 15). The dots represent individual females; the black line represents the mean; the colored lines represent the standard deviation. Unpaired *t* test; ***: *P* ≤ 0.001. D) Proportion of females that laid a batch of eggs (SkitoSnack: *n* = 77; animal blood: *n* = 21). Fisher’s exact test; *: *P* ≤ 0.05. E) Fertility measured as *n* larvae per egg raft / female (SkitoSnack: *n* = 1; animal blood: *n* = 4). The colored dots represent individual egg rafts; the black line represents the median.

To investigate the impact of the SkitoSnack diet on a later generation of *Cx. pipiens pipiens*, the same experiments were repeated using 2- to 5-day-old females of the F_13_ generation. The observed feeding rates (Fig. 3A) were 80.5% in the SkitoSnack group and 76.0% in the animal blood group, resulting in a total of 167 SkitoSnack-fed and 38 animal blood-fed females. The effects of the SkitoSnack on mortality (Fig. 3B) followed a similar trend as previously observed in Fig. 1B [Log-rank (Mantel–Cox) test, *P* ≤ 0.01]. The median survival after feeding on SkitoSnack was 13 days, significantly lower than the median survival of the animal blood group (54 days). Similar to the fecundity values of the F_4_ generation, F_13_ females that fed on the SkitoSnack had significantly fewer eggs than those fed with animal blood [Fig. 3C; unpaired *t* test (*t* = 9.793; *df* = 28; *P* ≤ 0.001)]. The mean number of eggs was 22 in the SkitoSnack group and 135 in the animal blood group. Only 6.5% (*n* = 5) of females in the F_13_ SkitoSnack group laid a batch of eggs (Fig. 3D; Fisher’s exact test, *P* ≤ 0.05), of which only one egg raft produced viable larvae (Fig. 3E). Unfortunately, the single egg raft from a SkitoSnack-fed female resulted in only 3 larvae that died before pupation.

### The Effects of the SkitoSnack on *Aedes aegypti*

**Fig. 4.**
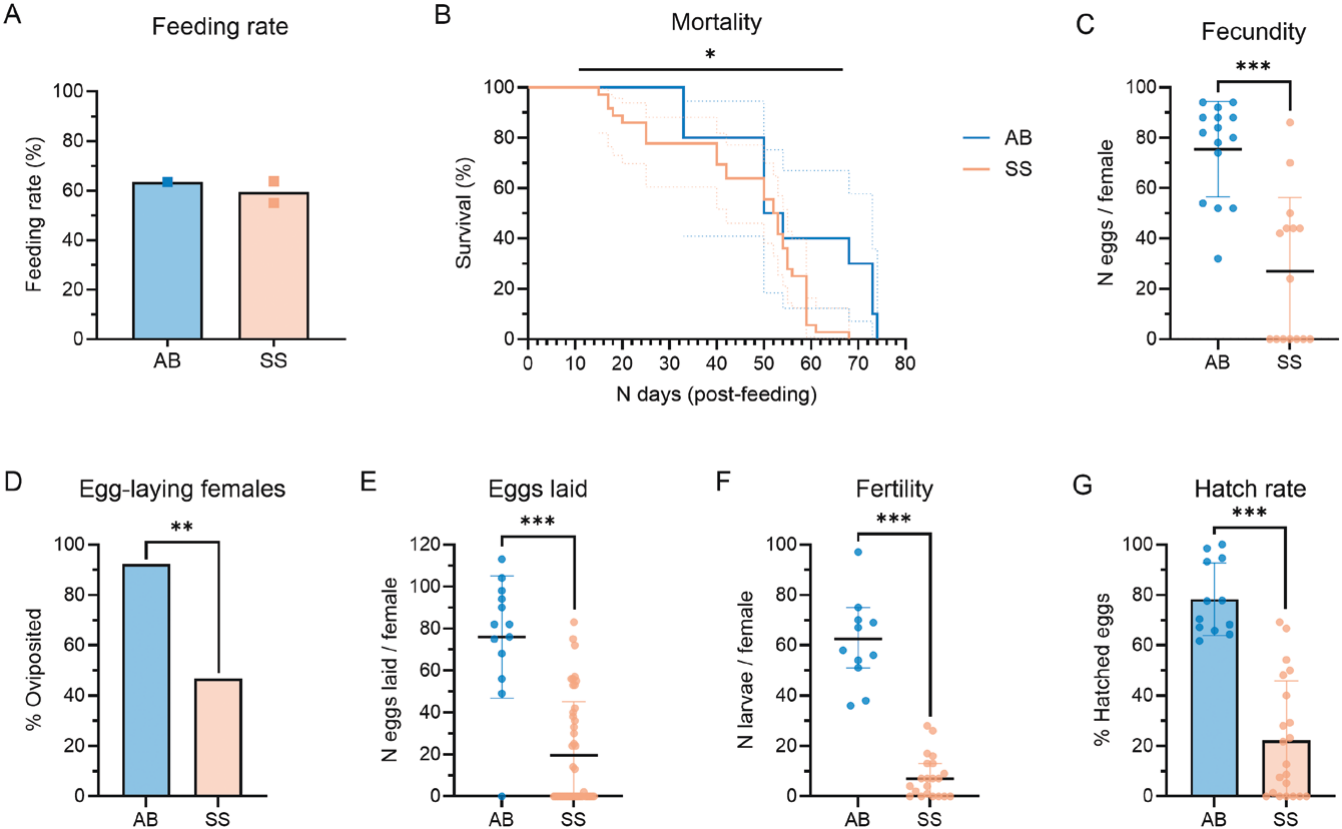
Feeding, survival, and reproduction of *Ae. aegypti* fed with SkitoSnack (SS) or animal blood (AB). A) Feeding rates of females offered the SkitoSnack (*n* = 107) or animal blood (*n* = 52). The bars represent the mean feeding rate and the squares represent a single feeding event. B) Mortality of fed females (SkitoSnack: *n* = 36; animal blood: *n* = 10). The dotted lines represent the 95% confidence intervals. Log-rank (Mantel–Cox) test; *: *P* ≤ 0.05. C) Fecundity measured as the total number of eggs dissected per female (SkitoSnack: *n* = 15; animal blood: *n* = 15). The dots represent individual females; the black line represents the mean; the colored lines represent the standard deviation. Mann–Whitney test; ***: *P* ≤ 0.001. D) Proportion of females that laid eggs (SkitoSnack: *n* = 45; animal blood: *n* = 13). Fisher’s exact test; **: *P* ≤ 0.01. E) Number of eggs laid per female (SkitoSnack: *n* = 45; animal blood: *n* = 13). The dots represent individual females; the black line represents the mean; the colored lines represent the standard deviation. Mann–Whitney test; ***: *P* ≤ 0.001. F) Fertility measured as *n* larvae per female (SkitoSnack: *n* = 21; animal blood: *n* = 12). The colored dots represent individual females; the black lines represent the median with 95% CI. Mann–Whitney test; ***: *P* ≤ 0.001. G) Hatch rate measured as the proportion (%) of hatched eggs. Mann–Whitney test; ***: *P* ≤ 0.001. The colored dots represent individual females; the colored lines indicate the standard deviation.

To determine if the intolerance of the SkitoSnack observed in *Cx. pipiens pipiens* is species-specific, we investigated the effects of the SkitoSnack on the life history of *Ae. aegypti* mosquitoes. *Ae. aegypti* females (7–10 days old) were provided with the SkitoSnack or animal blood as a control. The feeding rates were similar between the 2 treatment groups: 63% (animal blood) and 59% (SkitoSnack) (Fig. 4A). No mortality occurred in either feeding group during the first 14 days after feeding, and both feeding groups had similar median mortality (SkitoSnack: 52.5 days; animal blood: 52 days) (Fig. 4B). However, statistical analysis showed that there was a slightly higher risk of mortality in females fed with SkitoSnack compared to those fed with animal blood [Log-rank (Mantel–Cox) test, *P* ≤ 0.05]. When the ovaries were dissected, we observed that the SkitoSnack-fed females had significantly fewer eggs than the control females (Fig. 4C; Mann–Whitney test, *P* ≤ 0.001). The mean (± SD) egg counts were 27 ± 29 and 75 ± 19 in the SkitoSnack and animal blood groups, respectively. Overall, half of the SkitoSnack-fed females did not produce any viable eggs (*n* = 7/15) (Supplementary Fig. S2). The proportion of ovipositing females was also significantly lower (Fig. 4D; Fisher’s exact test, *P* ≤ 0.01). Almost all the females fed with animal blood laid eggs (92.3%), whilst roughly half of those fed with SkitoSnack laid eggs (46.7%). Fewer eggs were laid in the SkitoSnack group (Fig. 4E) with a mean (± SD) of 20 ± 26 eggs laid per female compared to 76 ± 29 mean eggs laid by females in the control group (Mann–Whitney test, *P* ≤ 0.001). SkitoSnack-fed females produced significantly fewer larvae than those fed with animal blood (Fig. 4F; Mann–Whitney test, *P* ≤ 0.001). The median number of larvae per female was 5 and 63 in the SkitoSnack and animal blood groups, respectively. All eggs in the control group hatched within the first 2 days of submergence in water, whereas most of those in the SkitoSnack group hatched on the third day (91%; range: 2–6 days). All females that laid eggs in the control group produced viable larvae (100%), whereas only 71.4% of females in the SkitoSnack group that laid eggs produced larvae (Fig. 4G). The mean hatch rate was significantly lower in the SkitoSnack group compared to the control group (SkitoSnack: 22.2%; animal blood: 78.3%; Mann–Whitney test, *P* ≤ 0.001).

**Fig. 5.**
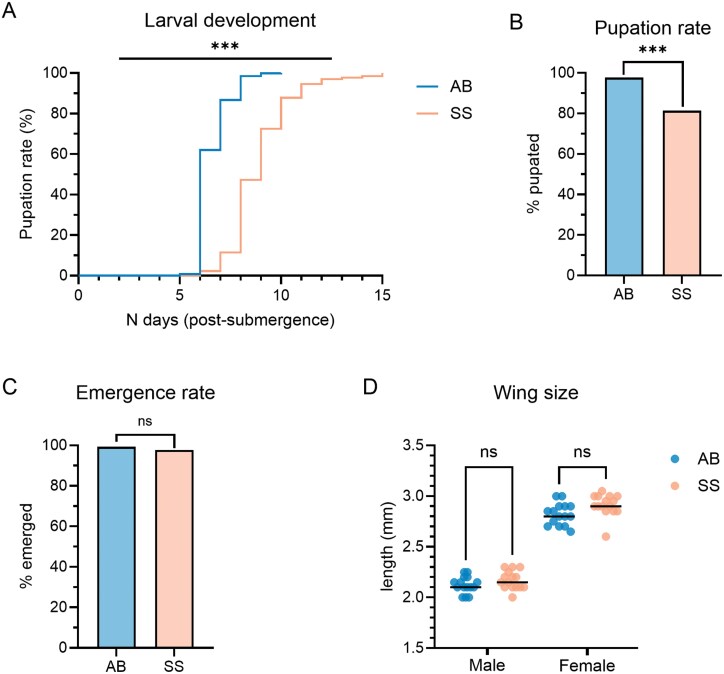
Fitness of *Ae. aegypti* offspring from parents fed with SkitoSnack (SS) or animal blood (AB). A) Rate of pupation (%) over number of days since eggs were submerged in water (SkitoSnack: *n* = 131; animal blood: *n* = 759). Log-rank (Mantel–Cox) test; ***: *P ≤ *0.001. B) Proportion (%) of pupae that molted from larvae (SkitoSnack: *n* = 161; animal blood: *n* = 777). Fisher’s exact test; ***: *P* ≤ 0.001. C) Emergence rate (%) from pupa to adult (SkitoSnack: *n* = 131; animal blood: *n* = 759). Fisher’s exact test; ns: non-significant. D) Wingspan (mm) of males and females (*n* = 15 per group). The colored dots represent individual values; the black lines represent the median. Kruskal–Wallis test; ns: non-significant.

Larvae in the SkitoSnack group took longer to develop than those derived from blood-fed females (Fig. 5A; Log-rank (Mantel–Cox) test, *P**** ***≤ 0.001). Almost all larvae in the animal blood group emerged into pupae (97.7%), whereas the probability of pupation was significantly lower in the SkitoSnack group (81.4%) (Fig. 5B, Fisher’s exact test; *P* ≤ 0.001). The median time to pupation was 6 and 9 days in the animal blood and SkitoSnack groups, respectively. The emergence rates from pupa to adult (animal blood: 99%; SkitoSnack: 98%) were not significantly different between the 2 feeding groups (Fig. 5C; Fisher’s exact test, *P* > 0.05). Likewise, the wingspans of the adult offspring were not significantly different [Fig. 5D; Kruskal–Wallis test, *P* > 0.05 (males), *P* > 0.05 (females)]. The mean wingspans were 2.2 and 2.1 mm for males and 2.9 and 2.8 mm for females in the SkitoSnack and animal blood groups, respectively. Longevity was not evaluated in the adult offspring.

## Discussion

Here we investigated if the SkitoSnack, an artificial diet developed for *Ae. aegypti* mosquitoes (Gonzales et al. 2018), could successfully produce a second generation of *Cx. pipiens pipiens* mosquitoes. Unfed female mosquitoes (F_4_) of 2 age groups were offered a meal of either the SkitoSnack or animal blood in a Hemotek feeding system. There was no difference in feeding rates for the younger age group (2–5 days old), but there was a slightly reduced willingness to feed on SkitoSnack in the older mosquitoes (7–10 days old). After feeding on the SkitoSnack, there was a significant increase in mortality compared to the control (animal blood) group. The median survival of females after feeding with SkitoSnack was only 4 days, roughly 10-fold lower than the control group. This rapid rise in mortality suggests that the SkitoSnack diet is toxic to recently fed mosquitoes, and death is likely to occur before the female is able to lay a batch of eggs.

A subset of fed *Cx. pipiens pipiens* females was dissected at 9 days post-feeding to measure egg counts, which were significantly reduced in the SkitoSnack group by 4-fold. Based on our observations of the eggs under the microscope, several females did not have fully matured eggs (based on the Christophers’ stages of development) (Christophers 1911). As the embryonic development of *Cx. pipiens pipiens* is roughly 2 days at 25 °C (Clements 1992), it is possible that the SkitoSnack does not contain sufficient nutrition for complete egg development. This could explain why SkitoSnack-fed females were less likely to lay a batch of eggs, as their egg development may not have been complete. Females were allowed to lay their eggs from 9 to 16 days post-feeding, which is a generous amount of time for laboratory colonies. Of the females in the SkitoSnack group that laid a batch of eggs, the fertility rates were significantly reduced by 3-fold. Overall, females fed with SkitoSnack had significantly reduced reproductive outcomes than those fed with animal blood.

In contrast, we observed similar life history outcomes in the offspring of both treatment groups. Larval development time from hatching to pupation was shorter in the SkitoSnack group by a median difference of 1 day. The pupation rate was not significantly different but the rate of adult emergence was marginally lower in the SkitoSnack group. As a proxy for body size, the wingspans of the SkitoSnack group were not significantly different from the control group. The longevity of the SkitoSnack group was also not significantly different. We hypothesize that the differences in larval development time and wingspan of the 2 feeding groups are due to the different numbers of larvae in the rearing pans. Rearing pans from the SkitoSnack group contained fewer larvae than the animal blood group (38 vs. 164 median larvae). It is therefore possible that larvae from the SkitoSnack group had more space and less crowding, allowing for faster and larger development than larvae from the animal blood group. Nonetheless, based on these results, the adult offspring of SkitoSnack-fed females had similar life history traits (fitness and longevity) as the control group.

Compared to the younger generation (F_4_), the older generation (F_13_) had a much higher feeding rate in both treatment groups. While we normally observe variable feeding rates with this colony, this higher willingness to feed may be due to the older generation being better adapted to the feeding methods used to maintain the colony. The survival rates after feeding on both the SkitoSnack and animal blood were longer than in the younger generation, but nonetheless, a similar negative effect of the SkitoSnack was observed whereby roughly half of the females died shortly after feeding. The same reduction in fecundity induced by the SkitoSnack was observed in the F_4_ and F_13_ generations (38 vs. 30 mean eggs per female, respectively), but fewer females in the F_13_ generation laid a batch of eggs (only 6.5% compared to 21.2% in the F_4_ females). The low oviposition rate resulted in one productive egg raft which produced only 3 larvae. These larvae were unable to molt into pupae, so the remaining experimental outcomes could not be evaluated. Overall, the SkitoSnack resulted in similar or worsened outcomes in the F_13_ generation. These results demonstrated the importance of evaluating artificial diets using multiple generations reared at different times. Furthermore, the lack of productive F_14_ offspring raised on SkitoSnack suggests there is a need for an optimized, re-formulated SkitoSnack diet to rear *Cx. pipiens pipiens*.

To understand if the negative outcomes of the SkitoSnack observed in *Cx. pipiens pipiens* are species-specific, we tested the effects of the SkitoSnack on *Ae. aegypti*. Compared to *Ae. aegypti* fed with animal blood, we found a minor effect of the SkitoSnack on the survival of fed females, but no mortality occurred within the first 2 weeks post-feeding. As the SkitoSnack was originally formulated for *Aedes* mosquitoes, we, therefore, suspect that the current composition and/or ratio of ingredients are toxic to *Cx. pipiens pipiens*. Surprisingly, we observed a significant reduction in fecundity and oviposition in SkitoSnack-fed *Ae. aegypti* similar to what we observed in *Cx. pipiens pipiens*. The study of Gonzales et al. observed that fecundity, oviposition rates, and wing lengths of *Ae. aegypti* maintained on SkitoSnack over 20 generations were not significantly different from their bovine blood-fed control colony (Gonzales et al. 2018). However, only the fecundity and oviposition of the 11th generation of SkitoSnack-reared mosquitoes were measured. It is therefore possible that the SkitoSnack-reared colony also exhibited significant differences in egg numbers and hatch rates in the initial stages of rearing. Moreover, an additional point to consider in the study of Gonzales et al. was the use of defibrinated blood, which can lead to fewer eggs laid by *Ae. aegypti* compared to fresh vertebrate blood (Dias et al. 2018). Another study, which investigated the effects of SkitoSnack on the life history of 3 *Ae. aegypti* laboratory colonies (UGAL, ROCK, and Liverpool), found that the first SkitoSnack feeding (F_0_) slightly diminished fecundity in 2 of the tested colonies (UGAL and Liverpool) (Kandel et al. 2020). In addition, they observed that while fecundity was reduced in the first feeding of the UGAL strain (F_0_), this difference was no longer observed after 30 generations of rearing with SkitoSnack (Kandel et al. 2020). The observed differences between our *Ae. aegypti* (Paea strain) and those used in previous studies may simply be due to variations between the colony strains. Although it may take several generations of feeding to improve the reproductive outcomes of SkitoSnack-fed females, we observed a significant reduction in the reproductive fitness of *Ae. aegypti* compared to the other studies, discouraging any further use of SkitoSnack for rearing this colony.

Compared to the original study by Gonzales et al. (2018), the methodology used here for preparing and handling the SkitoSnack meal had only minor differences. Most of the SkitoSnack reagents were obtained from the same manufacturer except for the BSA, ATP, NaHCO_3_, and KCl. While it would have been ideal to use ingredients obtained from the same manufacturers as the original study, we used products that were already available in-house. These ingredients may have contained trace substances responsible for the observed differences between these studies. Double-distilled water was used to re-suspend the dry SkitoSnack ingredients in the original study, whereas we opted for sterile Milli-Q water. Our study used the Hemotek system for feeding while theirs used water-jacketed feeders. Gonzales et al. placed engorged females in “breeding chambers” (50 ml conical tubes) with damp paper for egg deposition (suitable for *Aedes* mosquitoes), whereas the *C.* females in this study were placed in cages and given bowls of water to lay eggs directly on the water surface. Despite these minor methodological differences, it is more likely that the SkitoSnack formulation should be tailored to *Cx. pipiens pipiens* mosquitoes in order to reduce the negative impacts on survival and reproduction. *Cx. pipiens pipiens* are primarily ornithophilic (bird-biting) while *Ae. aegypti* and *Ae. albopictus* typically prefer feeding on humans and to a lesser extent on other mammals. An important determinant of host attractiveness may be due to the specific serological components required for egg production, as it is known that different blood sources can significantly impact mosquito reproductive outcomes [reviewed elsewhere (de Swart et al. 2023)]. Blood components such as the erythrocyte-packed cell volume, mean cell hemoglobin, serum protein (g/dl^−1^), serum albumin (g/dl^−1^), and serum triglycerides (g/dl^−1^) are fundamentally different between mammalian and avian species (Scanes et al. 2022). For future investigations of the SkitoSnack as a blood meal replacement for *Cx. pipiens pipiens*, a good starting point would be to re-formulate the SkitoSnack to match avian blood parameters. It would also be interesting to understand why the original SkitoSnack formula can induce mortality in one species but not another.

The significant loss of life, fecundity, and fertility in females fed with the SkitoSnack was a major bottleneck for the use of this artificial meal for rearing *Cx. pipiens pipiens* mosquitoes. Although we investigated the effects of the SkitoSnack in 2 different generations (F_4_ vs. F_13_), a limitation of this study was that only 1 subsequent generation was followed up. It would be interesting to monitor how the effects of rearing a *Cx. pipiens pipiens* colony exclusively on the SkitoSnack would affect the long-term fitness and reproduction over multiple generations. If overcoming the initial loss of life and reproductive fitness is the only major bottleneck, then it is possible that the SkitoSnack could be used to sustain multiple generations of *Cx. pipiens pipiens* without the need for animal blood. Another limitation of this study is that feeding volume (engorgement) was not quantified, possibly missing lower engorgement rates that caused the loss of reproductive capacity in SkitoSnack-fed females. A future study pinpointing the ingredient/s responsible for the high mortality, altering the ingredient ratios or supplementing the formula to improve the fecundity and fertility outcomes, and testing the modified SkitoSnack over several generations, would be the ideal next steps forward. Yet, based on the results observed in this study, the current SkitoSnack diet is not a viable alternative to vertebrate blood for rearing high numbers of *Cx. pipiens pipiens* over a single generation. Further optimizations of the SkitoSnack formula will be needed to prevent toxicity and improve the reproductive outcomes in *Cx. pipiens pipiens*.

## Supplementary Material

ieaf022_suppl_Supplementary_Figures_S1-S2
