# Supplementary material for: The artificial meal SkitoSnack does not support reproduction in Culex pipiens (Diptera: Culicidae) mosquitoes
Source: J Insect Sci. 2025 Apr 25;25(2):17. doi: 10.1093/jisesa/ieaf022 (PMC12023163; doi:10.1093/jisesa/ieaf022)
Supplement: ieaf022_suppl_Supplementary_Figures_S1-S2 [file ieaf022_suppl_supplementary_figures_s1-s2.docx]

###
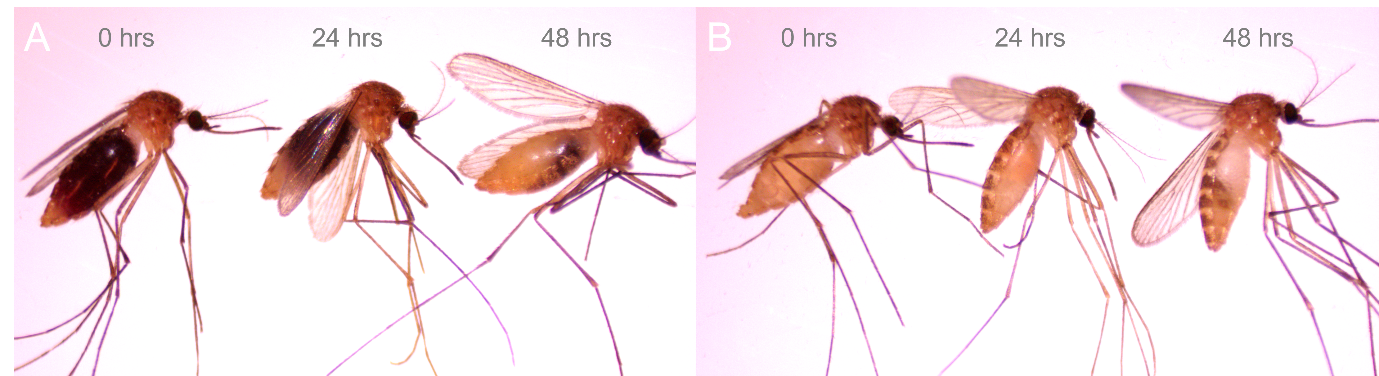
 Supplemental Fig. S1. Digestion of chicken blood (A) and SkitoSnack (B) in *Culex pipiens pipiens* at 0, 24, & 48 hours (hrs) post-feeding.

###
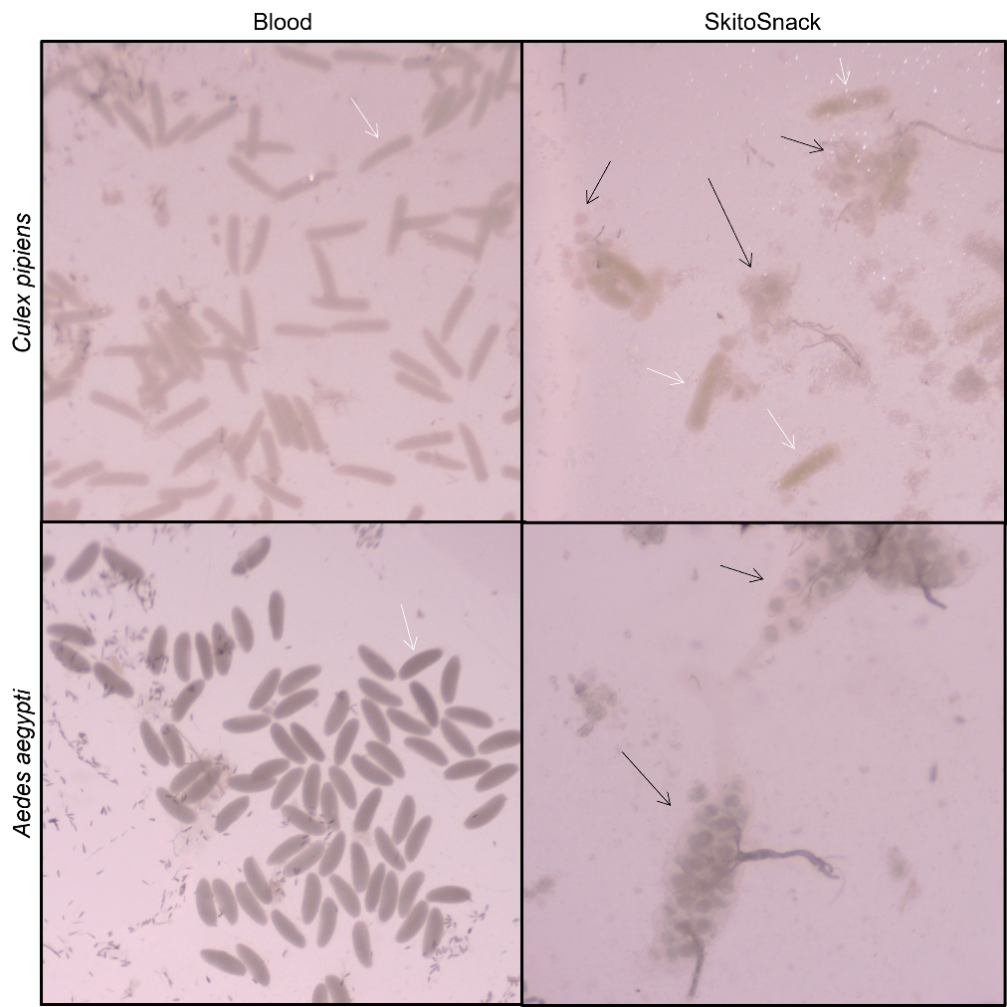
 Supplemental Fig. S2. Egg dissections of F_4_ *Culex pipiens pipiens* (top row) and *Aedes aegypti* (bottom row) fed with blood (left panels) or SkitoSnack (right panels). After feeding, females were given 9 days (*C. pipiens pipiens*) or 10 days (*A. aegypti*) to develop their eggs. Most *C. pipiens pipiens* fed with SkitoSnack had some fully matured (developed) eggs, but they often presented with a mixture of developed and underdeveloped eggs (top right panel). The *A. aegypti* fed with SkitoSnack either presented with fully matured eggs (as in the bottom left panel) or underdeveloped eggs (bottom right panel). The black arrows point to underdeveloped eggs and the white arrows indicate fully developed eggs, based on the Christophers’ stages of egg development.
